# Hepatic Epithelioid Angiomyolipoma: A Case Report

**DOI:** 10.7759/cureus.102476

**Published:** 2026-01-28

**Authors:** Nhan Hien Phan, Ngoc Trung Le, Dang Khanh Do, Thi Luan Dao, Thai Binh Nguyen

**Affiliations:** 1 Radiology, Hanoi Medical University Hospital, Hanoi, VNM; 2 Radiology, Hanoi Medical University, Hanoi, VNM; 3 Pathology, Hanoi Medical University Hospital, Hanoi, VNM; 4 Pathology, Hanoi Medical University, Hanoi, VNM

**Keywords:** hepatic epithelioid angiomyolipoma, hypervascular liver tumor, liver angiomyolipoma, liver tumor, pecoma

## Abstract

Hepatic epithelioid angiomyolipoma (HEAML) is a rare mesenchymal tumor belonging to the family of perivascular epithelioid cell tumors (PEComas), a group of mesenchymal neoplasms characterized by the proliferation of distinctive perivascular epithelioid cells expressing both melanocytic and smooth muscle markers. Because of its hypervascular nature and nonspecific imaging features, HEAML can closely mimic other hypervascular hepatic lesions, including hepatocellular carcinoma, hepatic adenoma, and focal nodular hyperplasia, making accurate preoperative diagnosis challenging. A 42-year-old woman was incidentally found to have a hepatic mass in the left lobe during a routine health examination. Abdominal ultrasonography revealed a relatively homogeneous hypoechoic lesion with well-defined margins and mild bulging of the liver capsule, containing thin internal echogenic strands. Magnetic resonance imaging demonstrated a mild signal drop on out-of-phase images compared with in-phase images, suggesting the presence of microscopic fat. Following contrast administration, the lesion showed strong heterogeneous arterial enhancement with punctate and linear intratumoral vessels, followed by prolonged enhancement in the portal venous and delayed phases (“wash-in, slow-out”). Early enhancement of a draining vein connected to the left portal vein branch was also observed. The patient underwent surgical resection. Histopathological examination revealed epithelioid cells with clear cytoplasm arranged around thin-walled blood vessels, without necrosis or significant mitotic activity. Immunohistochemical analysis showed positivity for HMB45, Melan-A, and smooth muscle actin, and negativity for HepPar-1, cytokeratin, and CD117, confirming the diagnosis of HEAML. HEAML is a rare hepatic tumor in which the integration of imaging findings and immunohistochemical analysis is essential for achieving an accurate diagnosis and guiding appropriate clinical management.

## Introduction

Hepatic epithelioid angiomyolipoma (HEAML) is a rare mesenchymal tumor of the liver composed of three components in variable proportions, including abnormal blood vessels, adipose tissue, and smooth muscle cells [1]. Owing to histologic heterogeneity and the frequently fat-poor nature of the lesion, preoperative diagnosis remains challenging [2]. The epidemiology of HEAML has not been clearly established; however, most hepatic angiomyolipomas are detected as solitary lesions in middle-aged women, with a proportion of cases occurring in association with tuberous sclerosis complex [3].

Although HEAML was previously considered a tumor with low malignant potential, recent evidence suggests that postoperative recurrence and distant metastasis can occur [4,5]. In clinical practice, HEAML is frequently misdiagnosed as other hypervascular hepatic tumors, particularly hepatocellular carcinoma (HCC), because of substantial overlap in both imaging and histopathologic features [6]. On magnetic resonance imaging, both lesions typically demonstrate strong heterogeneous arterial enhancement and may exhibit washout in the delayed phases [7]. Histologically, the presence of epithelioid cells with nuclear atypia, trabecular architecture, and carcinoma-like morphology can further contribute to misinterpretation as HCC [3].

Importantly, the therapeutic strategies for these entities differ substantially. HCC may be managed with tumor ablation, hepatic resection, liver transplantation, transarterial chemoembolization, or systemic therapies [8], whereas HEAML is generally managed with active surveillance or surgical resection, depending on imaging characteristics and histopathologic features suggestive of malignant potential [9]. Therefore, accurate differentiation between these two lesions is essential for determining appropriate treatment strategies prior to intervention or surgery.

Herein, we report a case of HEAML located in the left hepatic lobe that was diagnosed, surgically resected, and confirmed by histopathologic and immunohistochemical examination at our institution.

## Case presentation

A 42-year-old woman was incidentally found to have a mass in the left hepatic lobe during a routine health examination at another hospital five years earlier. The lesion was managed with periodic follow-up without intervention. The patient presented to our hospital because of a recent onset of epigastric discomfort and a sensation of fullness. On admission, physical examination findings were unremarkable. Laboratory tests, including complete blood count, liver function tests, serum alpha-fetoprotein (AFP), AFP-L3, and protein induced by vitamin K absence or antagonist-II (PIVKA-II), were all within normal limits. Serologic markers for hepatitis B and C virus infection were negative (Table 1). 

**Table 1:** Laboratory blood test results ALT: alanine aminotransferase; AST: aspartate aminotransferase; AFP: alpha-fetoprotein; PIVKA-II: protein induced by vitamin K absence or antagonist-II

Test	Result	Unit	Reference range
Red blood cell count (RBC)	3.66	Trillion/Litre	(4 - 5.4)
Hemoglobin (Hb)	129	g/L	(120 - 160)
Hematocrit (HCT)	0.34	L/L	(0.37 - 0.46)
White blood cell count (WBC)	9.10	Gigalitres	(4 - 10)
Neutrophils (%)	59.0	%	(45 - 75)
Lymphocytes (%)	29.3	%	(20 - 45)
Monocytes (%)	6.00	%	(0 - 8)
Eosinophils (%)	1.4	%	(0 - 8)
Basophils (%)	0.40	%	(0 - 2)
Neutrophils (absolute)	5.4	G/L	(1.8 - 7.5)
Lymphocytes (absolute)	2.66	G/L	(0.8 - 4.5)
Monocytes (absolute)	0.55	G/L	(0 - 0.8)
Eosinophils (absolute)	0.13	G/L	(0 - 0.8)
Basophils (absolute)	0.04	G/L	(0 - 0.1)
Platelet count (PLT)	320	G/L	(150 - 450)
Total bilirubin	3.4	µmol/L	(< 15)
Direct bilirubin	1.5	µmol/L	(< 3.4)
AST	42	U/L	(< 35)
ALT	40	U/L	(< 35)
AFP	1.80	ng/mL	(< 10)
AFP-L3	<0.5	%	(< 10)
PIVKA-II	10.00	mAu/mL	(< 40)

**Figure 1:**
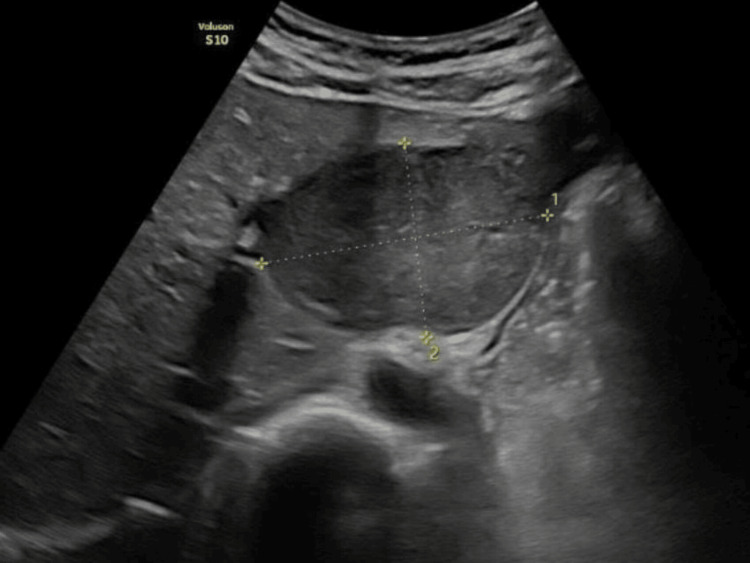
Abdominal ultrasonography demonstrating a relatively homogeneous hypoechoic mass in the left hepatic lobe, with a well-defined capsule and clear demarcation from the surrounding liver parenchyma.

Abdominal ultrasonography demonstrated a relatively homogeneous hypoechoic mass measuring 66×45 mm with well-defined margins and mild outward bulging of the liver capsule. Thin internal echogenic strands were observed within the lesion. No evidence of compression or invasion of the portal vein, hepatic veins, or adjacent organs was identified (Figure 1).

**Figure 2:**
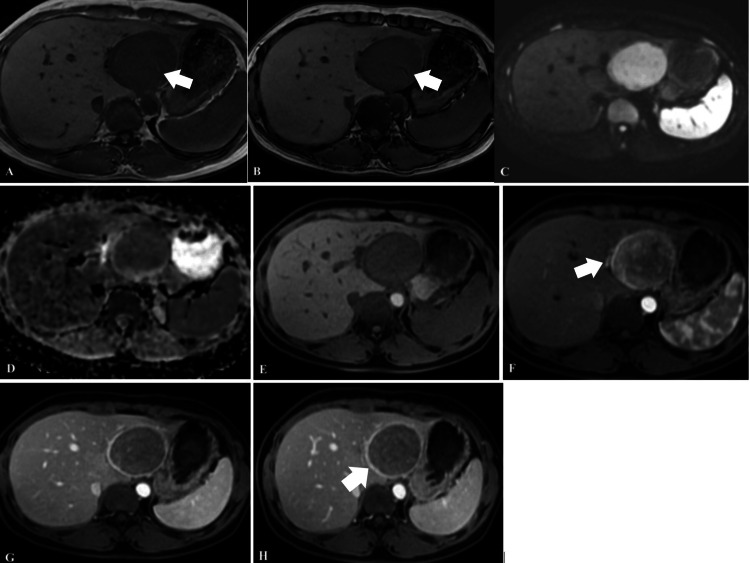
Magnetic resonance imaging findings A, B) T1-weighted in-phase and out-of-phase images show signal drop within the lesion on the out-of-phase sequence compared with the in-phase sequence, suggesting the presence of microscopic fat (arrowhead); (C, D) The lesion demonstrates diffusion restriction on diffusion-weighted imaging (DWI), with relatively homogeneous apparent diffusion coefficient (ADC) values compared with the surrounding liver parenchyma; (E-H) Dynamic contrast-enhanced MRI reveals strong, heterogeneous arterial-phase enhancement with intratumoral dilated abnormal vessels and early visualization of draining veins in continuity with the portal vein (arrowhead), followed by washout in the portal venous and delayed phases. The lesion is encapsulated, showing marked peripheral enhancement with persistent enhancement in the delayed phase (arrowhead).

Magnetic resonance imaging revealed a lesion with low signal intensity on T1-weighted images and subtle signal loss on out-of-phase images compared with in-phase images, suggesting the presence of microscopic fat. The lesion demonstrated diffusion restriction on diffusion-weighted imaging and corresponding apparent diffusion coefficient maps. After administration of gadolinium-based contrast, the tumor showed strong heterogeneous enhancement in the arterial phase, with punctate and linear intratumoral vessels. Early enhancement of a draining vein connected to the left branch of the portal vein was also observed. Peripheral enhancement surrounding the lesion persisted during the arterial, portal venous, and delayed phases. Delayed washout was noted in the portal venous and delayed phases. The lesion caused outward bulging of the liver capsule but showed no invasion of the portal vein, hepatic veins, or adjacent liver parenchyma. No additional hepatic lesions, extrahepatic abnormalities, or enlarged lymph nodes at the hepatic hilum were detected (Figure 2).

**Figure 3:**
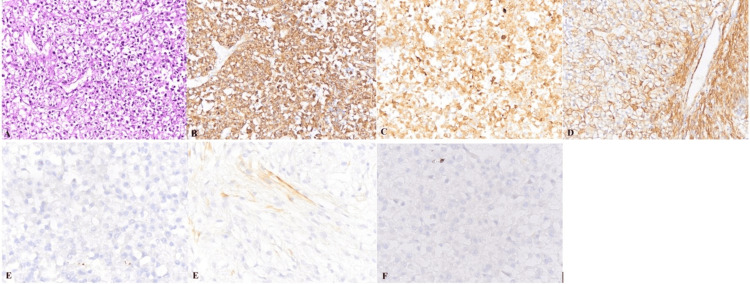
Histopathological and immunohistochemical findings (A) Hematoxylin and eosin (H&E) staining shows epithelioid cells with clear cytoplasm arranged around thin-walled blood vessels, without evidence of necrosis or atypical mitotic figures; (B-D) Immunohistochemistry is positive for HMB-45, Melan-A, and smooth muscle actin (SMA); (E-G) Immunohistochemistry is negative for HepPar-1, cytokeratin (CK), and CD117 (x100)

Based on the imaging findings, HCC was suspected; the multidisciplinary team decided to proceed with surgical resection after a thorough evaluation of the liver parenchyma and the future liver remnant, without performing a preoperative biopsy. Histopathological examination revealed epithelioid cells with clear cytoplasm arranged around thin-walled blood vessels, without evidence of necrosis or significant mitotic activity. Immunohistochemical staining was positive for HMB45, Melan-A, and smooth muscle actin, and negative for HepPar-1, cytokeratin, and CD117. These findings confirmed the diagnosis of HEAML (Figure 3). Postoperatively, the patient was followed up monthly with ultrasonography and tumor marker measurements for three months and subsequently every three months for one year. No complications or tumor recurrence were observed during the follow-up period.

## Discussion

HEAML is an extremely rare subtype of hepatic angiomyolipoma. Available data suggest that HEAML predominantly affects middle-aged women and usually presents as a solitary, well-circumscribed, round lesion, consistent with the findings in our case [10]. In the present patient, the tumor was incidentally detected in the left hepatic lobe in the setting of a non-cirrhotic liver with normal serum AFP levels, which helped to reduce the likelihood of HCC, a malignancy that is commonly associated with chronic liver disease and elevated AFP levels [11].

The clinical presentation of HEAML is often nonspecific, as most patients are asymptomatic or present with vague symptoms such as epigastric discomfort, right upper quadrant pain, abdominal fullness, low-grade fever, or weight loss [10]. Imaging modalities, particularly magnetic resonance imaging, play a crucial role in guiding the diagnosis. On MRI, subtle signal loss on out-of-phase images suggests the presence of microscopic fat and represents an important diagnostic clue, although many HEAMLs contain little or no detectable fat [3,12]. After contrast administration, HEAML typically demonstrates strong arterial enhancement with delayed washout in the portal venous and delayed phases, producing a characteristic “wash-in, slow-out” pattern, which differs from the classic “wash-in, wash-out” pattern of HCC (Figure 2) [11,13]. The presence of punctate and linear intratumoral vessels during the arterial phase reflects the rich vascularity of the tumor and is consistent with the angiogenic nature of HEAML [12]. In addition, persistent peripheral enhancement surrounding the lesion across all contrast phases is a suggestive imaging feature, reflecting abundant peripheral tumor vasculature rather than the true capsule enhancement commonly seen in HCC [12].

Histopathologically, HEAML is characterized by epithelioid tumor cells with eosinophilic cytoplasm arranged around thin-walled blood vessels. These cells typically have round to polygonal cytoplasm, large round nuclei, and prominent nucleoli, while adipose tissue may be minimal or absent. The predominance of epithelioid cells is considered a key diagnostic feature, although the exact proportion required for diagnosis remains controversial. Immunohistochemically, positivity for HMB45, Melan-A, and smooth muscle actin represents the characteristic immunophenotype of perivascular epithelioid cell tumors [14]. Strong expression of melanocytic markers such as HMB45 and Melan-A is particularly useful for differentiating HEAML from HCC and hepatic adenoma, which are typically negative for these markers [14]. The absence of epithelial markers such as HepPar-1 and cytokeratin further supports a non-hepatocellular origin. Another important differential diagnosis was metastatic epithelioid gastrointestinal stromal tumor (GIST), which was excluded in the present case based on negative CD117 immunoreactivity [14,15].

Regarding management, surgical resection followed by regular postoperative surveillance is generally recommended for large tumors, lesions with atypical imaging features, or cases in which histopathologic findings suggest malignant potential, such as necrosis, increased mitotic activity, or vascular invasion [9].

## Conclusions

HEAML is a rare hepatic tumor characterized by epithelioid cells with variable mesenchymal components. Certain imaging features, including microscopic fat, a “wash-in, slow-out” enhancement pattern, peripheral rim enhancement, and prominent intratumoral vessels, may suggest the diagnosis. Definitive diagnosis, however, relies on histopathologic and immunohistochemical evaluation, particularly the expression of melanocytic markers.
